# Static and dynamic changes of natural head position improve vertical eye canting of patients with non-syndromic asymmetric facial deformities after orthognathic surgery

**DOI:** 10.3389/fsurg.2025.1678943

**Published:** 2026-01-06

**Authors:** Wei Sun, Tianyu Zhao, Yuxin Tang, Xiang Li, Siyong Gao, Guangsen Zheng

**Affiliations:** Guangdong Provincial Key Laboratory of Stomatology, Department of Oral and Maxillofacial Surgery, Guanghua School of Stomatology, Sun Yat-sen University, Guangzhou, Guangdong, China

**Keywords:** orthognathic surgery, facial asymmetry, eye asymmetry, natural head position, three-dimensional cephalometry

## Abstract

**Objective:**

By combining static and dynamic methods of obtaining natural head position, this study examined whether compensatory head tilt in patients with non-syndromic asymmetric facial deformities contributes to of pseudo-“vertical eye canting”.

**Methodology:**

Patients with non-syndromic asymmetric facial deformities patients and symmetric dento-maxillofacial deformity who met the inclusion and exclusion criteria were divided into observation and control groups. Static photographs were measured to analyze the static head position of the subjects in each follow-up period; dynamic natural head positions of the subjects in different states in each period were recorded by posture sensors for dynamic analysis. Intergroup and intragroup changes were analyzed with Mann–Whitney U-test and one way ANOVA.

**Results:**

The static natural head position of the observation group at 1 year after surgery was significantly different from that of the pre-surgery period, whereas each dynamic natural head position at all follow-up periods after surgery was significantly different from dynamic natural head position of the pre-surgery period. The range of dynamic head position in the rolling direction was smaller in the observation group than in the preoperative period at 6 months and 1 year after surgery, but the dynamic head position range in the rolling direction was not significantly different in the control group at the different follow-up stages.

**Conclusions:**

Changes in natural head position (NHP) following orthognathic surgery were associated with improved stabilization of head posture in roll orientation and reduced pseudo-eye canting in non-syndromic asymmetric facial deformities (NSAFD) patients. Clinical Trial Number KQEC-2021-58-01.

## Introduction

Vertical asymmetry of eye position, known as eye canting, is often thought to be an anatomic ocular displacement of developmental origin. Mild asymmetry of the horizontal position of the whole eye and vertical position of the ocular adnexa has been found by previous studies, and these mild deformities can often be improved by soft-tissue surgeries such as blepharoplasty and oculoplastic surgery (1, 2). Severe vertical discrepancy in levels of orbits, also known as orbital vertical dysplasia, require craniofacial surgery for improvement (3, 4).

Besides, eye canting is often observed in syndromic and non-syndromic asymmetric facial deformities, and the eye canting of patients with non-syndromic asymmetric facial deformities (NSAFD) were found to be partly improved after orthognathic surgery in a study of static photographs. It is believed that natural head position (NHP) of the patients with NSAFD tilted preoperatively, and it was corrected postoperatively (5). However, this hypothesis that partly improvemt of eye canting appear after orthognathic surgery is controversial.

NHP is a standard position of the head in an upright head posture with the eyes focusing on a point in the distance at eye level (6). Studies often use static methods to measure NHP, such as radiography, 2D photographs and 3D imaging technique including stereophotogrammetry, laser scanning and facial radiopaque markings combined with cone beam computed tomography, which have shown good reproducibility (7, 8).

Measurement of the NHP is gradually gaining attention from physicians in various fields. Experienced orthodontists do not only refer to conventional 2D or 3D cephalometric measurements because of the anatomical variation of the intracranial references on which cephalometric measurements are based. In addition, studies have found that different NHP may result in different cephalometric measurements, even when the lateral profile are identical in the photographs (9). For prosthodontics, prosthodontists are also aware that changes in head position can affect the judgement of restoration of the teeth (10).

For orthognathic surgery, the NHP is important to virtual surgical planning (11). Before jaw bone segments movement, an appropriate midsagittal plane or midline must be defined, which is sometimes difficult in facial asymmetric patients. A widely accepted method for establishing the midsagittal plane involves first defining a horizontal plane, most commonly the Frankfurt horizontal plane, which is then used to derive the midsagittal plane (12). There are several methods to define the facial midline, such as the perpendicular bisector of the bilateral eyebrows, the line passing through the nasion and philtrum, and the perpendicular bisector of bilateral pupils (13–15). For the midsagittal plane, the prevailing methods of establishment include a plane through the cranial median landmark or a plane perpendicular to the line of bilateral symmetry landmarks. Apparently, the eyes are most important anatomical landmark for defining the midsagittal plane and facial midline, but bilateral height of eyes can be changed as the NHP altered after orthognathic surgery. Thus, how NHP and eye canting change after orthognathic surgery must be taken into consideration when performing the virtual surgical planning of facial asymmetric patients.

Instead of a single, static position, NHP, as a body posture, is a small rangle of positions. Thus, static photographing might not be accurate enough for measuring NHP. Studies of Fränkel and Lundström et al. suggested that NHP is actually a dynamic concept and should be recorded dynamic methods (16). Dynamic measurement devices were gradually used to measure dynamic changes in NHP. Murphy et al. designed a device capable of continuously recording single-axis angles for recording dynamic head positions (17). Usumez and Orhan achieved measurement and transfer of dynamic NHP data using a smaller device (18).

However, current clinical studies of NHP suffer from homogenization of assessment, short follow-up time, and lack of control. Therefore, this study focuses on these issues in a one-year prospective cohort study aiming to figure out how NHP changes after orthognathic surgery in the patients with NSAFD combined with eye canting by using dynamic and static measurements. The results might be taken into consideration for the orthognathic surgeons when defining the midplane during virtual surgical planning in the patients with NSAFD.

## Materials and methods

### Patient inclusion, exclusion and grouping

From Jan. 2020 to Jan. 2022, the consecutive patients with dento-maxillofacial deformities in Hospital of Stomatology, Sun Yat-sen University who were to receive conventional bimaxillary orthognathic surgery and met the inclusion criteria of observation group or control group were enrolled in this study.

Inclusion criteria for patients of observation group: (1) Patients presented non-syndromic facial asymmetry (the menton point must be deviated greater than 3 mm from the perpendicular bisector of the bipupillary line) and eye canting. The direction of menton point deviation is toward the side of the lower eye with the head in the NHP. (2) The angle of eye canting (the angle between the line through their bilateral pupils and the horizontal line) of the patients in the NHP are greater than 3.0° by means of both static and dynamic measurement. Inclusion criteria for patients of control group: (1) Patients presented nonsyndromic dento-maxillofacial deformities and the menton point must be deviated less than 1 mm. (2) The angle of eye canting of the patients in the NHP are less than 1.0° by means of both static and dynamic measurement. Exclusion criteria for patients: (1) Patients with a history of craniomaxillofacial/neck trauma or surgery; (2) Patients diagnosed with cleft lip and palate or other syndromic congenital anomalies; (3) Patients diagnosed with torticollis or scoliosis; (4) Patients diagnosed with vestibular dysfunction; (5) Patients refused to participate in this study.

**Figure 1:**
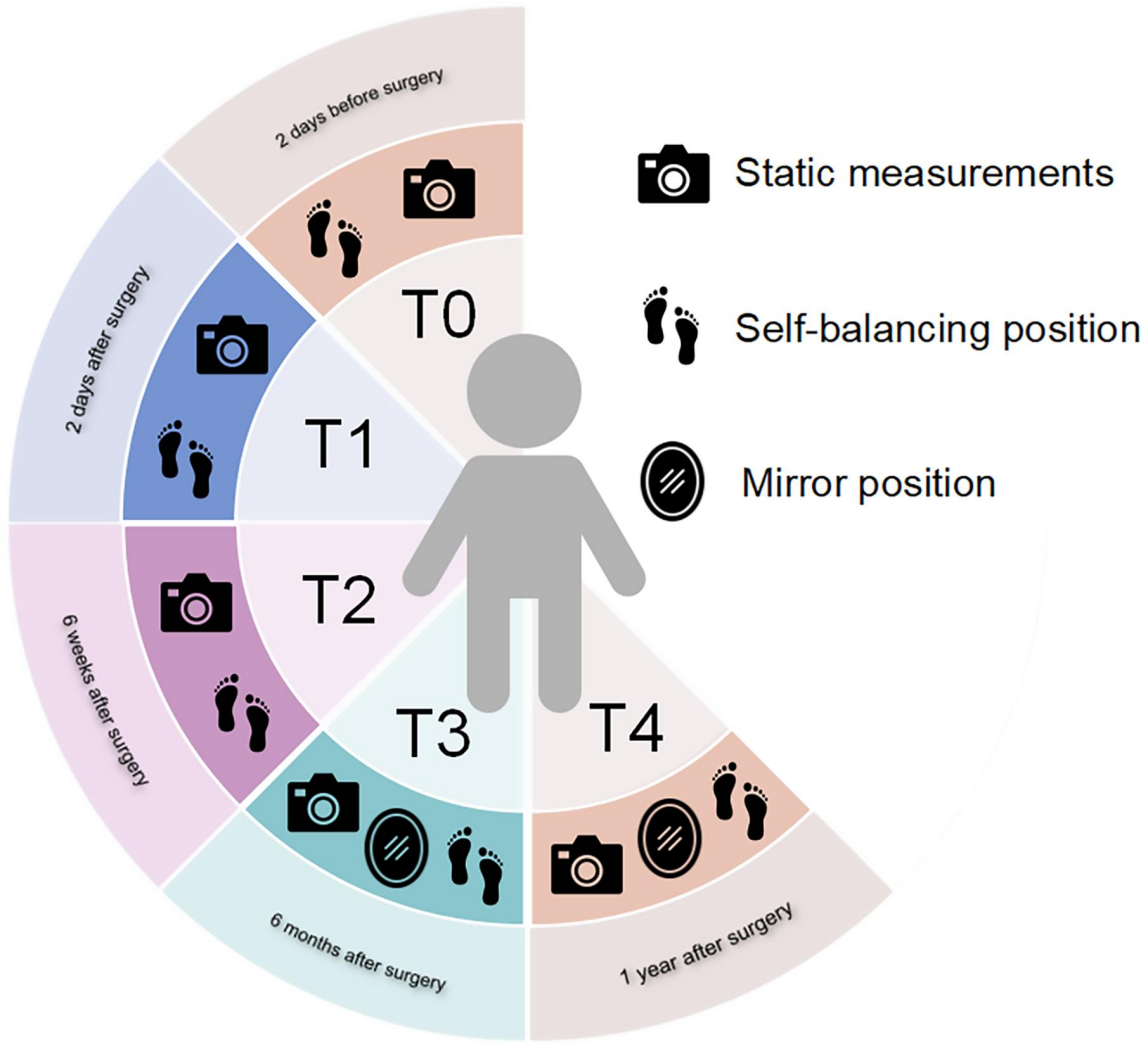
Follow-up plan.

In this study, five follow-up time points were selected, including T0: 2 days before surgery; T1: 2 days after surgery; T2: 6 weeks after surgery; T3: 6 months after surgery and T4: 1 year postoperatively. Data recording of dynamic NHP and static NHP in self-balance position (SBP) was performed during each follow-up visit, and dynamic NHP in mirror position (MP) recording was added at T3 and T4. Patients' static and dynamic measurements at T0 were used as baseline data and for patient inclusion exclusion. Figure 1 show the patient's follow-up plan of measurement.

### Static measurements

Static measurements of the NHP were taken from photographic measurement. The patients were first asked to relax head and neck before photographing. Then the patients were asked to stand in front of the white background, to maintain a straight back, and to look straight ahead with a relaxed facial expression with fully open eyes and gently closed lips. A digital camera (Canon EOS90D digital camera, 32.5 Megapixel; Canon Japan, Tokyo) with a 100-mm lens and a ring flash, was used in the 12 o'clock position. The height of the lens of the camera was adjusted on the tripod to match the eye level of the subject. The digital camera took 3 static full-face images. Between photographs, patients were asked to walk around and relax their heads and necks. The camera had an aperture setting of F6.3 and was at a standardized distance of 5 feet (1.5 m) from the subject.

### Dynamic measurement

**Figure 2:**
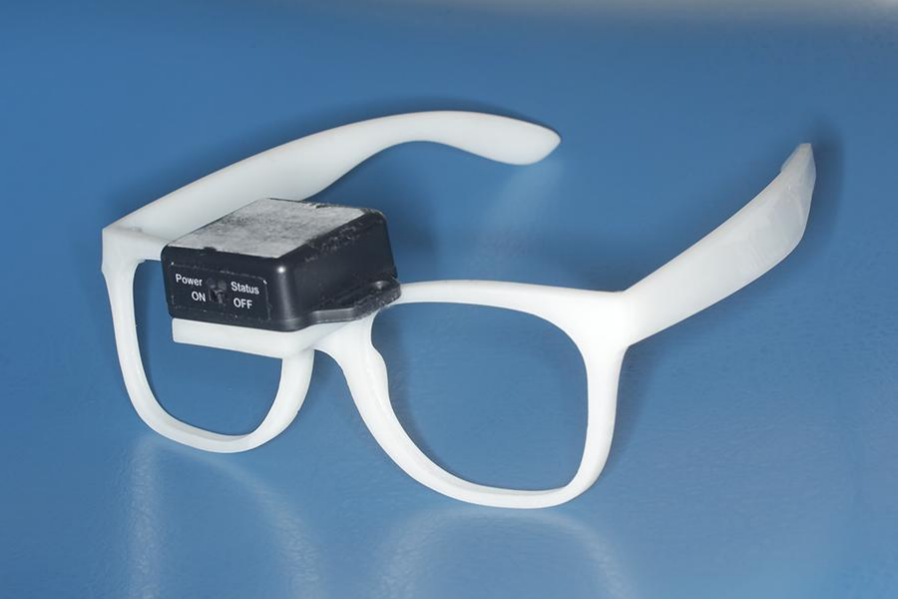
Instrument used for recording dynamic head position in study.

The instrument of dynamic measurement was an eyeglass frame with axial sensor (BWT901CL, Axial Sensor, Wit-Motion China, Shenzhen), which can measure the pitch, yaw and roll of the head (Figure 2). The axial sensor carried a gyroscope which measured 51.5 mm × 36.1 mm × 15 mm and was fixed in the microplatform of the nose bridge of the eyeglass frame. The total weight of the apparatus, including the 3 axial sensor and the eyeglasses frame, was 21.67 g. Prior to formalizing the dynamic data acquisition, we assessed the reliability of the dynamic NHP measurement equipment using an electronic protractor with manufacturer-confirmed accuracy. The instrument has been calibrated by the manufacturer and must undergo repeatability testing before each independent measurement session. The specific reliability assessment procedure is as follows: Prior to measuring, the sensor is attached to the manufacturer-provided electronic goniometer. The goniometer is then gradually opened from 0° to 90° in 10° increments. Both the sensor and goniometer acquire corresponding readings at each position, which are recorded by the host computer for consistency analysis. Should the resulting *R*-value fail to meet the specified requirements, the instrument must be returned to the manufacturer for recalibration.

**Figure 3:**
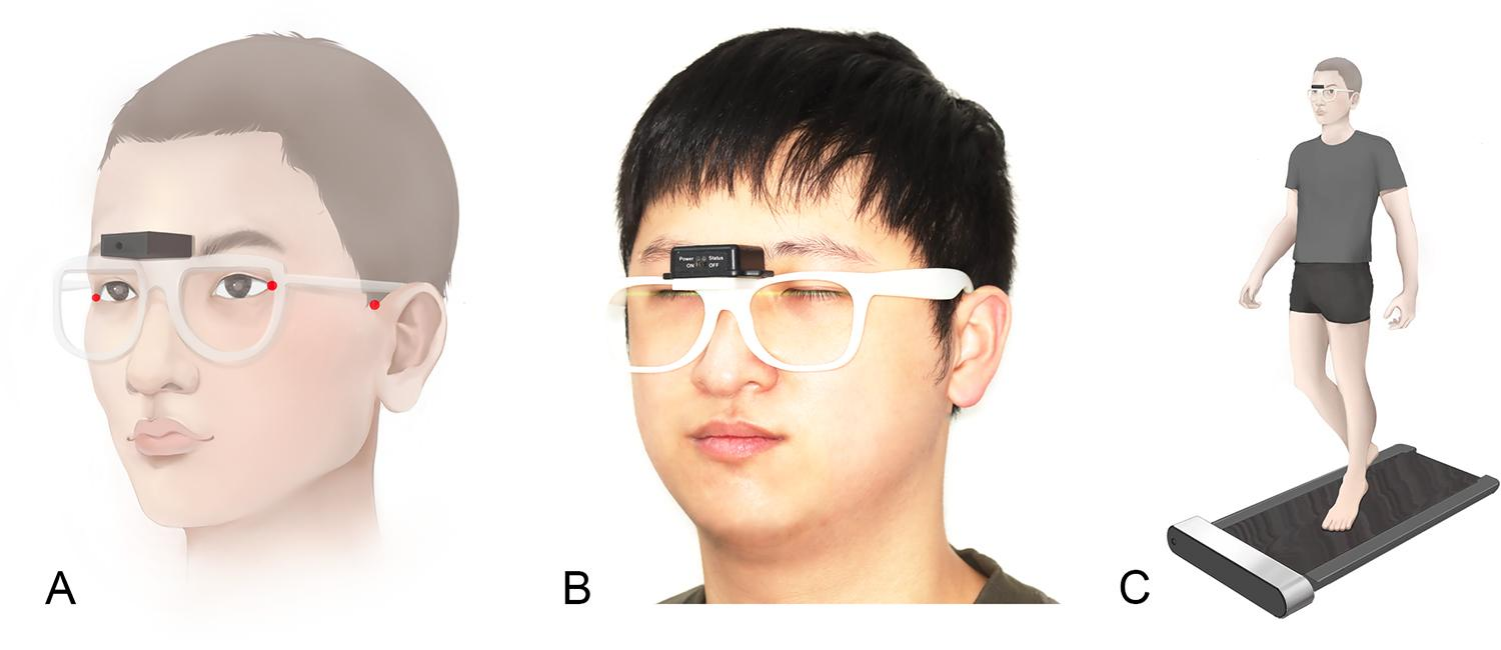
Reference landmarks for dynamic measurements and data collection methods. [**(A,B)** Reference landmarks and leveling of head position; **(C)** Methods of collecting dynamic natural head positions].

Subjects acclimated to the instruments for 5–10 min and determine their comfortable walking speed on the treadmill. Before the measurement, with the aid of a laser level meter, we made the line through the bilateral lateral canthus and the junction of the outer helix and the temporal skin of the patients parallel to the horizontal plane when patients wear the instrument (Figure 3). Then they walked on a treadmill at a constant speed for 70 s each measurement as they wore the instruments (Figure 3). The dynamic NHP during walking of T3 and T4 was determined in two ways of each subject, one defined by the subjects own feeling of a natural head balance (self-balance position, SBP) and one by the subject looking straight into a mirror (mirror position, MP). The NHP of T0, T1 and T2 was determined in SBP. Between measurements, the subjects were asked to relax their necks, move around, and then reestablish the position. At each follow-up visit, each kind of dynamic NHP was measured four times for each subject.

**Figure 4:**
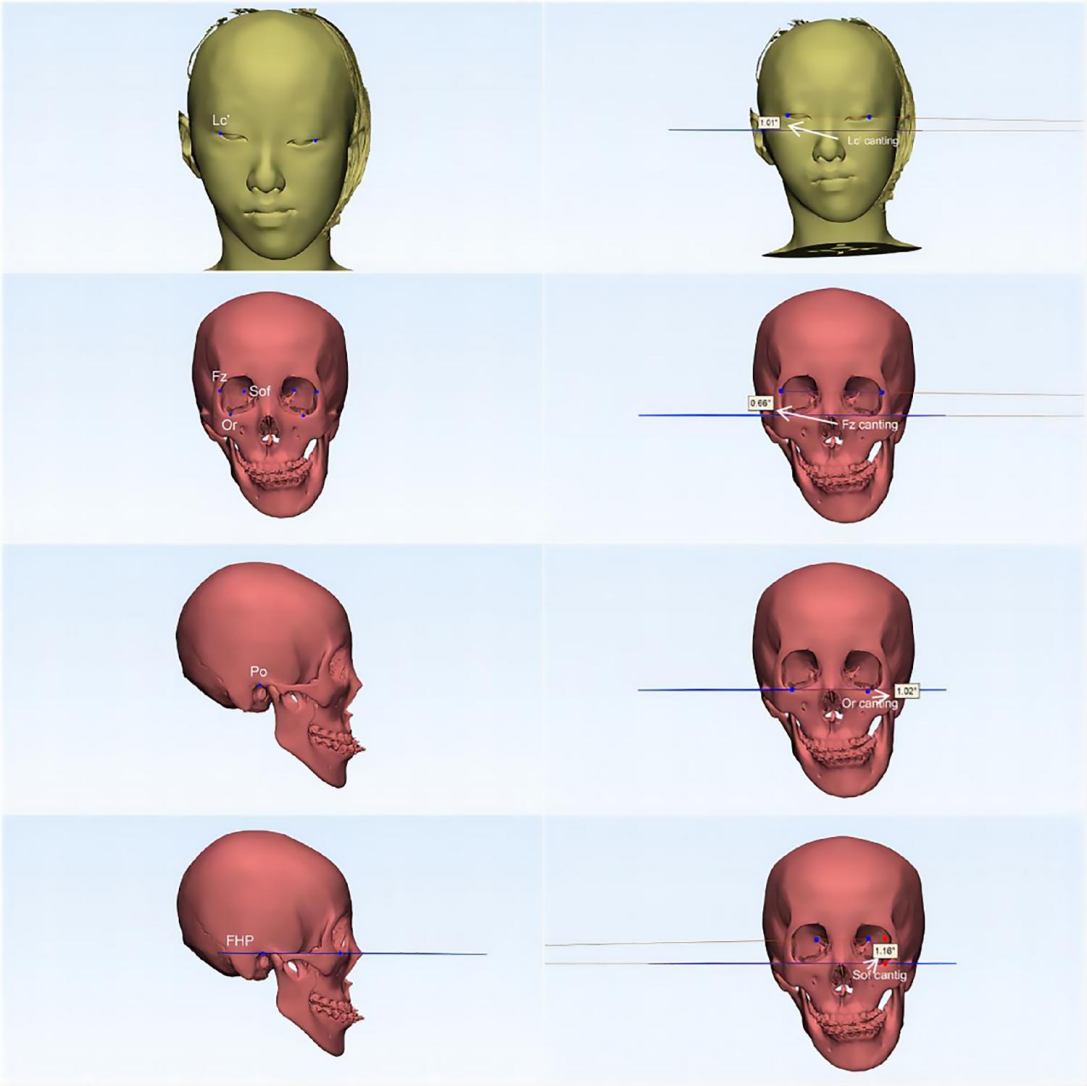
3d cephalometric assessment for exclusion of patients with asymmetric upper facial skeleton. (Po, Porion; Fz, Frontozygomatic suture; Sof, Superior orbital fissure; Lc', Lateral canthus; Or, Orbitale point; FHP, Frankfurt horizontal plane).

Preoperative CT images were taken in supine position with the use of a SOMATOM Force CT scanner (Siemens Healthineers, Erlangen, town in Bavaria, Germany). The images were stored in DICOM files, were opened and reconstructed with Mimics 21.0 software (Materialise, Leuven, Belgium) on a Windows system and the segmentation process was started. The threshold values of semiautomatic segmentation were from 226 to 3,071 Hounsfield units (HU) for the hard tissues and from −700 to 225 HU for the soft tissues (Figure 4). Reconstructed soft and hard tissue images were converted to the standardized stereolithography (STL) file format and transferred to the 3-matic Research 13.0 software (Materialise, Leuven, Belgium) for marking of landmarks, segmentation of anatomic areas and measurement of facial symmetry. In this study, the Frankfurt horizontal plane (FHP) was chosen as the reference plane, which was defined as the plane passing through the bilateral Po point and the right Or point, to determine the spatial location of each landmark of the craniofacial region. The researcher marked landmarks directly on the images reconstructed by the 3-matic Research 13.0 software and performed subsequent measurements. The landmarks, reference plane, reference lines, measurement values and specific measurement methods are presented in Figure 4 and Table 1.

**Table 1:** (A) three-dimensional landmarks, reference plane and reference lines. (B) Angular measurements.

(A) Landmarks (abbreviation)	Definition
Porion (Po)	Most upper point of meatus acusticus extemus
Frontozygomatic suture (Fz)	Most forward point of the frontozygomatic suture
Superior orbital fissure (Sof)	Most upper point of Superior orbital fissure
Lateral canthus (Lc')	Outer commissure point of the upper and lower eyelids
Orbitale point (Or)	Lowest point of the bony orbit
(A) Reference plane and lines	
Frankfurt horizontal plane (FHP)	Plane passing through the bilateral porion points and the right orbitale point
Lc' line	The line passing through the bilateral Lc' points
Or line	The line passing through the bilateral Or points
Fz line	The line passing through the bilateral Fz points
Sof line	The line passing through the bilateral Sof points
(B) Angular measurements	
Lc' canting (°)	Angle between the Lc' line and FHP
Or canting (°)	Angle between the Or line and FHP
Fz canting (°)	Angle between the Fz line and FHP
Sof canting (°)	Angle between the Sof line and FHP

### Statistical analysis

Data analysis was performed using the IBM SPSS software package for Windows (version 23.0; SPSS, Chicago, Illinois). Descriptive statistics were calculated as the quartiles of angle measurements. The normality of the reliability assessment data was evaluated by means of the Kolmogorov–Smirnov test and Quantile-Quantile plot. Reliability assessment of equipment was through Pearson or Spearman correlation analysis. Differences in NHP between periods in the same group were analyzed by Friedman test. Mann–Whitney U test were used to analyzed the differences in NHP between groups in the same period. The range of roll-oriented dynamic head position of the same group in different periods were analyzed by Friedman test. Dynamic NHP in MP and SBP in different periods, and the angle measurements of different groups were also analyzed by Mann–Whitney U test.

### Surgical reference and procedures

Based on a standardized surgical design protocol to minimize inter-observer variability, all patients in both groups underwent orthognathic surgery according to the following reference system: (1) The facial midline was defined as the perpendicular bisector of bilateral ocular landmarks, while the Frankfurt horizontal plane (FHP) was established using the unilateral Porion (Po) and bilateral Orbitales (Or) to ensure a horizontal bipupillary line. This approach aimed to ensure postoperative facial symmetry relative to the perpendicular bisector of the bipupillary line; (2) The maxillary and mandibular segments were aligned according to the final occlusal relationship; (3) The maxillomandible complex was repositioned sagittally to achieve an improved facial profile; (4) In the coronal plane, the maxillomandible complex was repositioned to bring the menton point close to the perpendicular bisector of the bipupillary line postoperatively. This design aimed to achieve postoperative facial symmetry relative to the perpendicular bisector of the bilateral ocular landmarks.

Guided by the surgical designs, patients underwent the Le Fort I osteotomy and bilateral sagittal split ramus osteotomies (SSRO) with or without maxillary segmentalization, anterior mandibular subapical osteotomy and genioplasty. Surgeries were carried out via an intraoral approach, using an electric saw. The occlusion was re-established with interocclusal wafers prior to fixation. Fixation of the maxilla was performed in a routine manner with titanium miniplates and screws applied to the piriform and zygomaticomaxillary buttresses. The SSRO technique used was the Hunsuck-Dal Pont modification of the Obwegeser sagittal split ramus osteotomy. Rigid fixation using titanium miniplates and screws was applied to the SSRO segments.

## Result

One patient in the observation group was loss to follow up. The final sample consisted of 20 volunteers and were divided into observative group (*n* = 10) and control group (*n* = 10), 20–25 years of age.

Spearmon's correlation analysis suggested a statistically significant correlation coefficient of *R* = 0.9952 (95%*CI* was 0.9943–0.9959) between the device measurements and the electronic protractor measurements at *α* = 0.05.

**Figure 5:**
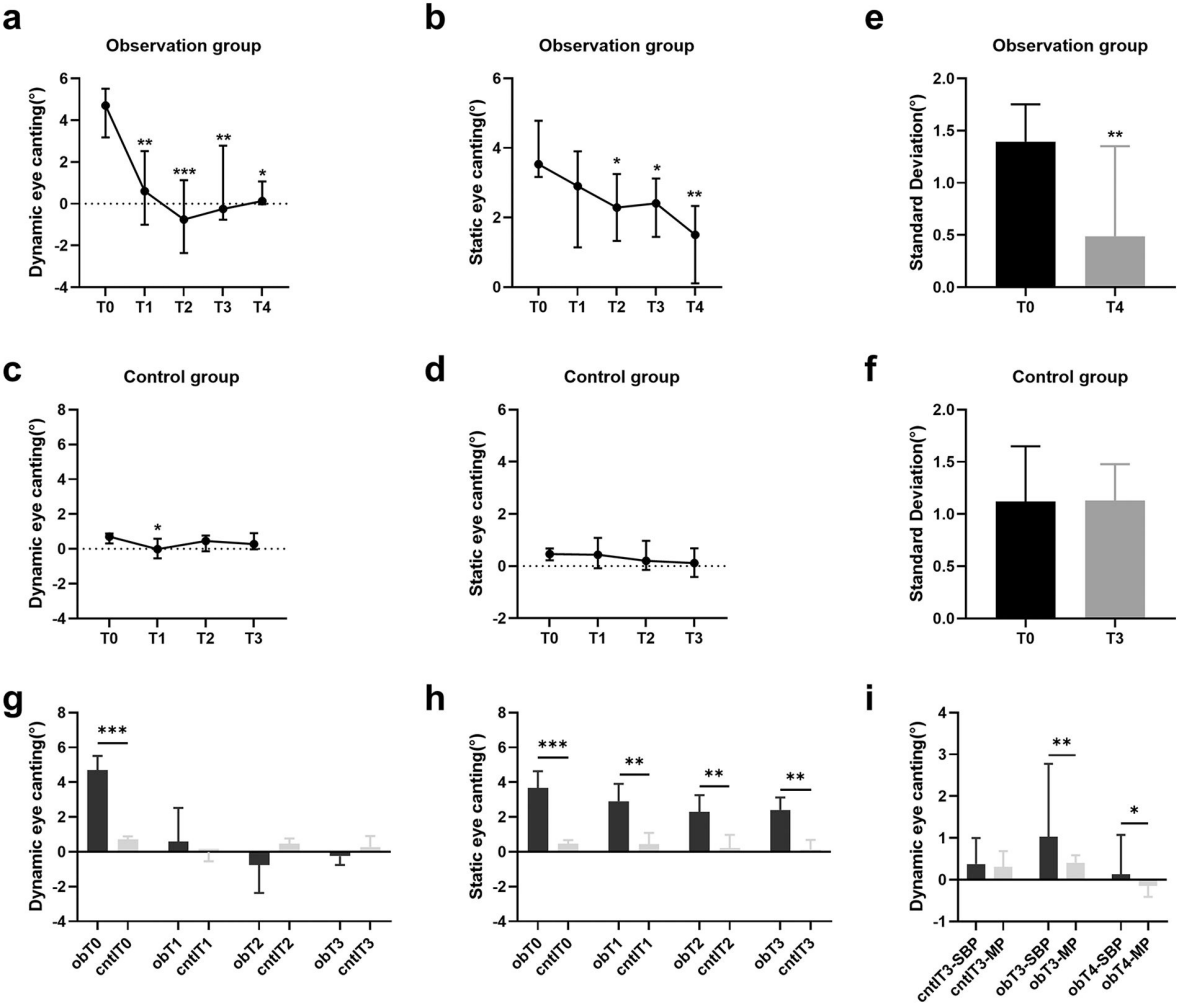
Preoperative and postoperative changes in natural head position. **(a)** Dynamic natural head position changes in the observation group. All data are expressed as median and quartile. *P < *0.05,* **P < *0.01,* ***P < *0.001* versus T0. (Friedman test). **(b)** Static natural head position changes in the observation group. All data are expressed as median and quartile. *P < 0.05, **P < *0.01* versus T0. (Friedman test). **(c)** Dynamic natural head position changes in the control group. All data are expressed as median and quartile. *P < *0.05* versus T0. (Friedman test). **(d)** Static natural head position changes in the control group. All data are expressed as median and quartile. (Friedman test) **(e)** Change in range of natural head position swings of observation group. All data are expressed as median and quartile. **P < *0.01* versus T0. (Paired samples Wilcoxon signed rank test) **(f)** Change in range of natural head position swings control group. All data are expressed as median and quartiles. (Wilcoxon signed-rank test). **(g)** Changes in differences between dynamic natural head positions. All data are expressed as median and quartile. ***P < *0.001* versus T0. (Mann Whitney test). **(h)** Changes in differences between static natural head positions. All data are expressed as median and quartile. ***P < *0.001*, **P < *0.01* versus T0. (Mann Whitney test). **(i)** Differences in dynamic natural head position between MP and SBP in the postoperative period. MP, mirror position; SBP, self-balance position; cntl:control group; ob, observational group. All data are expressed as median and quartile. **P < *0.01*, *P < *0.05* versus T0. (Paired samples Wilcoxon signed rank test).

**Figure 6:**
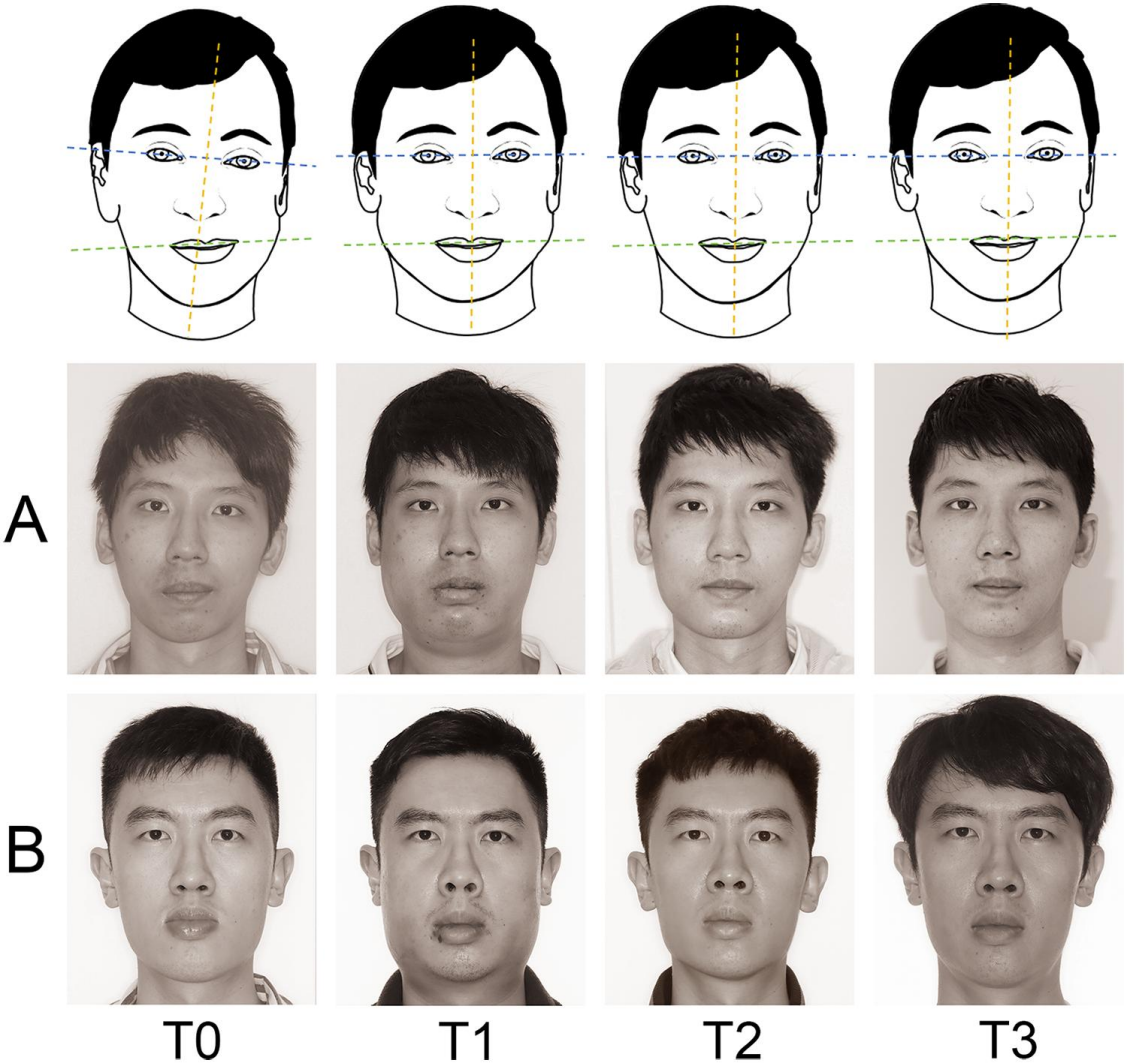
Changes of static natural head position. [**(A)** observation group; **(B)** control group].

Table 2 shows the descriptive data of each group for each follow-up period. Only in T4 the static NHP of observation group were significantly different from T0, whereas the dynamic NHP of all postoperative periods were significantly different from T0 (Figures 5a,b). In observation group, the range of roll-oriented dynamic NHP in T3 and T4 was reduced compared to T0, but in control group, the range of roll-oriented dynamic head positions in different follow-up periods were insignificantly different (Figures 5e,f). Dynamic NHP between two groups in preoperative periods were significantly different and were not significantly different in postoperative periods and there was a significant difference between the preoperative and postoperative static NHP in both groups (Figures 5g,h). Three dimensional cephalometry suggested that the degree of displacement of ocular soft and hard tissue landmarks was not significantly different (Supplementary Figure S1). Significant differences were observed between postoperative dynamic NHP in MP and SBP in the observation group but not in control group (Figure 5i and Table 3). Figure 6 shows the change in static head position of 1 patient in the observation group and 1 patient in the control group at each follow-up period.

**Table 2:** Descriptive data for dynamic and static measurements.

Natural head position	Distribution of observation group (*n* = 10)		Distribution of control group (*n* = 10)	
	static	dynamic	static	dynamic
T0	3.533 (3.167, 4.784)	4.705 (3.178, 5.510)	0.467 (0.225, 0.675)	0.708 (0.315, 0.887)
T1	2.900 (1.144, 3.903)	0.600 (−1.005, 2.523)	0.434 (−0.083, 1.083)	−0.015 (−0.549, 0.582)
T2	2.285 (1.326, 3.250)	−0.755 (−2.363, 1.128)	0.200 (−0.142, 0.971)	0.450 (−0.133, 0.761)
T3	2.405 (1.443, 3.125)	−0.245 (−0.690, 1.894)	0.117 (−0.418, 0.684)	0.378 (0.106, 0.999)
T4	1.500 (0.108, 2.333)	0.133 (−0.048, 1.073)	–	–

The values are shown as median (first quartile, third quartile).

**Table 3:** Dynamic measurements of MP and SBP.

Natural head position	Distribution of observation group (*n* = 10)			Distribution of control group (*n* = 10)		
	MP	SBP	*P*	MP	SBP	*P*
T3	0.407 (−0.577, 0.585)	−0.245 (−0.690, 1.894)	<0.01	0.310 (−0.345, 0.685)	0.378 (0.106, 0.999)	0.39
T4	−0.154 (−0.412, 0.210)	0.133 (0.048, 1.073)	<0.05	–	–	-

The values follow normal distribution and are shown as median (first quartile, third quartile).

MP, mirror position; SBP, self-balance position.

## Discussion

Asymmetry of the tissues around the eyes is common, and soft and hard tissue difference have been shown to play a role in facial asymmetry (2, 19). Trend of horizontal periocular asymmetry and slight lateral asymmetry of the eyelid, orbit, and eyebrow have been found by previous studies (1, 2). Studies on vertical asymmetry have demonstrated that patients can detect vertical asymmetry between bilateral eyelids and eyebrows, with discriminative thresholds of 2 mm and 3 mm, respectively (20, 21). Three-dimensional cephalometric measurements of periorbital soft and hard tissue landmarks in the patients included in this study suggested that there was no significant correlation between asymmetry of periorbital soft and hard tissue landmarks and asymmetry of ocular vertical position in patients with NSAFD. This result suggests that patients with NSAFD who have combined vertical ocular asymmetry do not show more significant anatomical asymmetry of the periocular tissues than patients with relatively symmetrical faces. Anatomical asymmetry of the periocular tissues is not the main causes of “eye canting” of ocular appearance in patients with NSAFD.

While anatomical factors alone may not fully account for ocular asymmetry in NSAFD, the potential role of functional adaptations becomes particularly relevant when considering how surgical interventions can alter body posture. The phenomenon of treatment leading to changes in body posture has been reported in breast and abdominal surgery. Studies of breast reduction and abdominal rectus plication abdominoplasty have found that patients present with changes in body posture after breast volume reduction or abdominal morphologic changes (22, 23). These changes were associated with body mass distribution, but were also thought to be due to surgery eliminating dissatisfaction with body image, reducing anxiety, and increasing self-esteem. A static photographic study confirmed that the tilted head position in NSAFD patients may functionally compensate for lower facial asymmetry, resulting in a bilateral ocular appearance with an elevated position on one side and a lowered position on the other, which appears to improve after orthognathic surgery (5). Combining dynamic and static measurement, our study further revealed a similar trend in head position after orthognathic surgery in these patients in the dynamic dimension. Following surgery, we observed concurrent improvements in orbital canting and head positioning, consistent with reduced compensatory mechanisms. These findings indicate an association between orthognathic surgery and reduction of pseudo-eye canting, potentially mediated through changes in compensatory head tilt. These patients who undergo orthognathic surgery to correct the lower 1/3 of the face may be able to achieve good facial symmetry without further ocular surgery.

To accurately quantify these postsurgical changes in craniofacial morphology and posture, three-dimensional analysis provides indispensable methodological support. Regarding three-dimensional analysis, CT-based 3D reconstruction enables comprehensive cephalometric assessment. While comparative studies between 2D and 3D cephalometry have yielded conflicting conclusions, and 3D techniques present limitations including higher radiation exposure and strong dependency on cranial positioning references (24), consensus acknowledges that 3D measurements overcome fundamental 2D limitations such as magnification errors and projective distortions. They provide superior quantification of asymmetric structures and facilitate integrated assessment of craniofacial morphology through linear/angular measurements, airway dynamics, volumetric changes, and muscular adaptations—all crucial for surgical planning and preoperative evaluation (25).

Within 3D craniofacial assessment, establishing a reliable coordinate system is fundamental, with the Natural Head Position serving as a critical extracranial reference. Studies have found that the NHP is stable and reproducible and can be used as an extracranial reference during treatment. The longitudinal serial studies of Cooke demonstrated that NHP is a reference line that has significant stability through 15 sequential years of observation (26–28). Registered natural head position (RNHP) and estimated natural head position (ENHP) are two basic methods to getting the NHP. RNHP is registered when the head of the patient is orientated to his or her NHP and a mark or a plumb line is used as a registration point in the radiographs or photographs. ENHP is obtained by rotating patient's conventional cephalograms or lateral facial photographs to their NHP under the judgment of an experienced specialist (29). Although the ENHP has the advantages of being patient-neutral and simple to perform over the RHNP, studies comparing the ENHP with the RNHP have found that the ENHP is affected by the morphology of the patient's chin and that inter-investigator variability affects the accuracy of the ENHP. Therefore, the RNHP, which is not subjectively influenced by the researcher, was chosen for this study. Modalities for guiding the patient to obtain a NHP include the MP and the SBP. MP is the head position obtained by the subject looking straight into a mirror. SBP is the head position determined by the subjects own feeling of a natural head balance. A study of stereophotogrammetry has suggested that the error of the NHP between these two methods is acceptable (8). However, some researchers have argued that MP is an unnatural, restricted head posture, and that the presence of a mirror is inducing because it presents a body image to the subject, and that SBP without any external reference is the most relaxed and natural position for the head (26, 30–33).

Therefore, we hypothesized that comparing MP and SBP would reveal effects of body image concern on NHP; thus, we assessed both conditions to explore this relationship in patients with NSAFD. We found a statistically significant difference in tilt angle between NHP obtained by MP and SBP and a smaller tilt angle in NHP obtained by MP, which supports the influence of body image concern on NHP. This finding tentatively suggests that mirror feedback might serve as a potential intervention for postural instability rehabilitation, though this requires validation, as in previous studies (34).

It is important to note, however, that NHP should not be conceptualized as a single static orientation, but rather as a dynamic equilibrium state. With more research on NHP, NHP was considered to be a small range of positions oscillating around the subject's mean NHP (MNHP). Thus, head posture is a dynamic concept and its recording should be dynamic and continuous (15). Previous studies have noted that the standard deviation of dynamic head position of the NHP in the population is between 1° and 2°, which was similar to our findings (16, 17). In addition we found that the range of preoperatively roll-oriented head position was greater than that after orthognathic surgery in patients with NSAFD combined vertically positional asymmetry of the eyes, which was not observed in patients with relatively symmetric face. The observed association between orthognathic surgery and improved dynamic NHP stability suggests a potential beneficial relationship, a result that is consistent with the hypothesis of Gian Maria Galeazzi et al. Their findings suggest that although increase of body sway is often the result of either sensory failure, both anxiety and body image concerns are negatively correlated with indexes of posturographic stabilization, which partly validates the sychopathological interference between perceived and actual balance disturbance (35).

This dynamic understanding of NHP necessarily raises questions about the adequacy of static measurements in capturing its full complexity. Differences have been found between the static NHP and the NHP during dynamic walking, and one of the explanations for this observation is that continuous measurements are able to measure the full range of positional oscillating around the subject's MNHP, whereas the static measurements of the head position may only be at one of the extremes of the above ranges (36). It has also been suggested that the walking process requires an adjustment of the head position to better obtain a view of subject's steps or the surroundings, and therefore there is a difference between the two NHP (37). Additionally various subjective factors during the recording of head position may affect the static NHP measurement. Our study found that dynamic measurement data can detect changes in NHP at an earlier follow-up period, and also suggests potential differences in dynamic and static NHP. This further suggests that the acquisition of dynamic NHP is indispensable in clinical practice and scientific research.

These limitations of static assessment underscore the importance of developing sophisticated dynamic measurement technologies for accurate NHP evaluation. Dynamic measurement devices have been widely used to measure NHP (17). Digital sensors have been recognized as having similar accuracy to laser scanning and have been suggested as a new paradigm for clinical measurement of NHP (38, 39). Serdar Usumez et al. recorded changes in pitch and roll by fixing two single-axis inclinometers symmetrically on each arm of a pair of eyeglasses (37). And the dynamic head position measured by the device was also found to have good repeatability through repeated measurements (18). The study of Murphy et al. showed that the inclinometer had no significant effect on the wearer's head position (17). The single multi-axis posture sensor used in this study was kept as light and small as possible. It was designed to be placed right in the middle of the glasses in order to avoid affecting the measurement of NHP in roll.

## Conclusion

Changes in Natural Head Position following orthognathic surgery were associated with improved stabilization of head posture in roll orientation and reduced pseudo-eye canting in NSAFD patients.

## Data Availability

The raw data supporting the conclusions of this article will be made available by the authors, without undue reservation.
