# Supplementary figures and images for: Static and dynamic changes of natural head position improve vertical eye canting of patients with non-syndromic asymmetric facial deformities after orthognathic surgery
Source: Front Surg. 2026 Jan 6;12:1678943. doi: 10.3389/fsurg.2025.1678943 (PMC12816371; doi:10.3389/fsurg.2025.1678943)

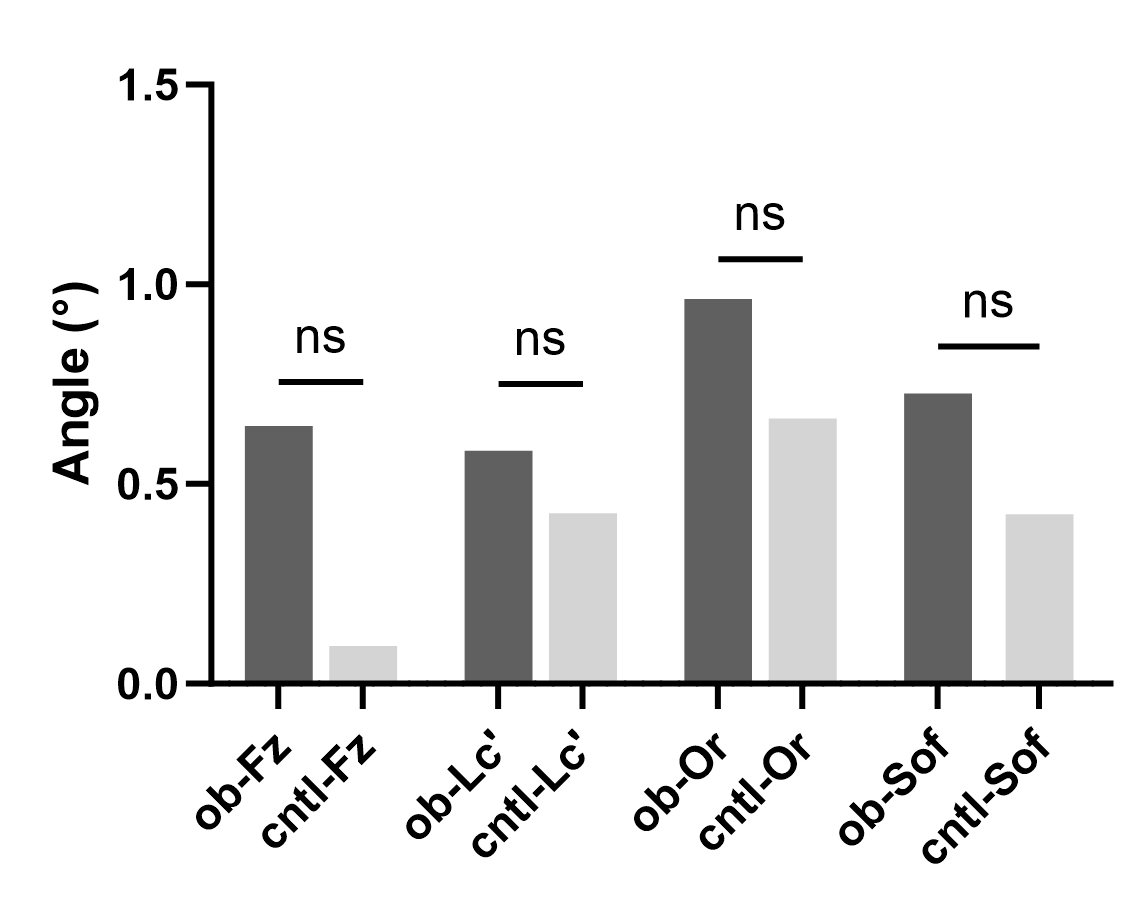

Supplement: Supplementary file 1 [file Image1.tif]
